# Supplementary material for: Wastepaper-Based Cuprammonium Rayon Regenerated Using Novel Gaseous–Ammoniation Injection Process
Source: Polymers (Basel). 2024 Aug 27;16(17):2431. doi: 10.3390/polym16172431 (PMC11397804; doi:10.3390/polym16172431)
Supplement: Supplementary file 1 [file polymers-16-02431-s001.zip › polymers-3084504-supplementary.pdf]

Supplementary of

Synthesis and Characterization of Cuprammonium Rayon Fibers from Short Rotational Leucaena's Prunings Using a Novel Gaseous-Ammoniation Injection Process

Sherif S. Hindi

Section 1. Synthesis of the Cuoxam R

Figure S1. The prototype-scaled synthesis of copper hydroxide [Cu $(OH)_2$]: **A)** copper sulphate ($CuSO_4.5H_2O$): a1) a commercial bag, a2) crystal aspect, a3) Its solution (5 %, wt/wt); B) sodium hydroxide (NaOH): b1) a commercial container, b2) its crystal aspect, b3) Its solution (1 %, wt/wt); C) : c1) the starting of mixing the copper sulphate (5 %, wt/wt) with the NaOH solution (1 %, wt/wt) with the same volumetric ratio (1:1), c2) progress reacting the $CuSO_4.5H_2O$ with the NaOH, c3) complete synthesis of the $Cu(OH)_2$, and c4) the sedimentation of the $Cu(OH)_2$ paste; D) the novel gaseous ammoniation injection process used to inject ammonia gas into the Cu $(OH)_2$: d1) pressurized injection of the gas in a special vessel; d2) the Cuoxam solution.

Section 2. The Universal Testing Machine

Figure S2. Universal testing machine: a) Instron, model 1193, and b) Preparing the rayon fibers samples for mechanical properties determinations.

Section 3. Fixation of the staple fibers-bundle's rayon in the Universal Testing Machine.

Figure S3. Schematic representative of fixing the staple fibers-bundle's rayon for measuring the tensile properties.

Section 1. Synthesis of the Cuoxam R

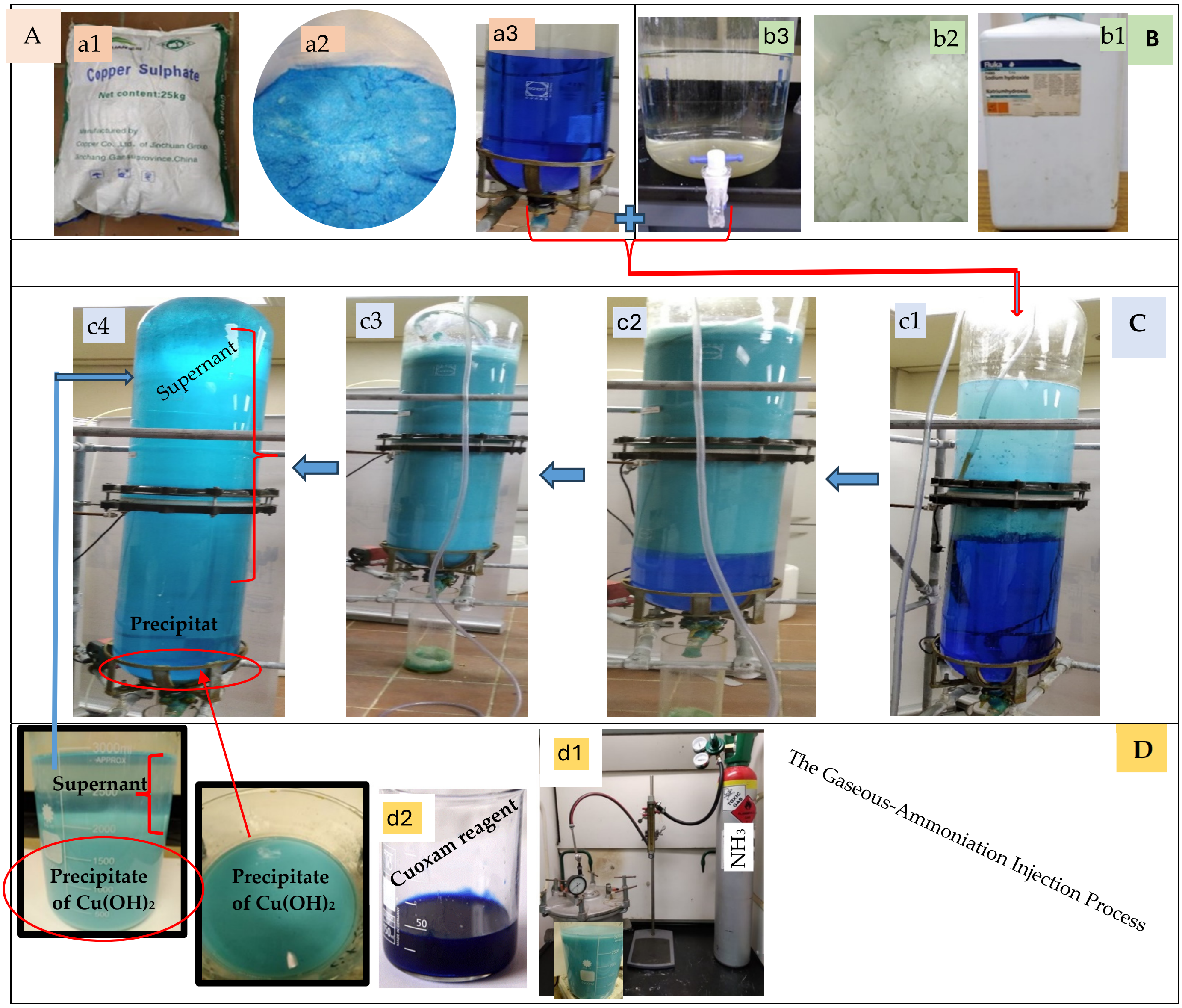

Figure S1. The prototype-scaled synthesis of copper hydroxide [Cu $(OH)_2$]: **A)** copper sulphate ($CuSO_4.5H_2O$): a1) a commercial bag, a2) crystal aspect, a3) Its solution (5 %, wt/wt); B) sodium hydroxide (NaOH): b1) a commercial container, b2) its crystal aspect, b3) Its solution (1 %, wt/wt); C) : c1) the starting of mixing the copper sulphate (5 %, wt/wt) with the NaOH solution (1 %, wt/wt) with the same volumetric ratio (1:1), c2) progress reacting the $CuSO_4.5H_2O$ with the NaOH, c3) complete synthesis of the $Cu(OH)_2$, and c4) the sedimentation of the $Cu(OH)_2$ paste; D) the novel gaseous ammoniation injection process used to inject ammonia gas into the Cu $(OH)_2$: d1) pressurized injection of the gas in a special vessel; d2) the Cuoxam solution.

Section 2. The Universal Testing Machine

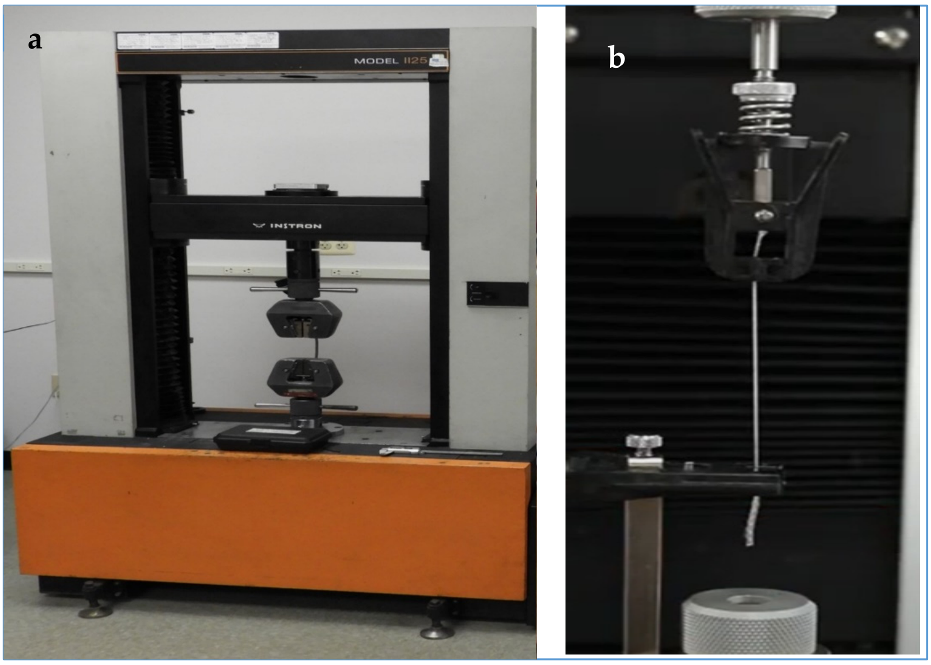

Figure S2. Universal testing machine: a) Instron, model 1193, and b) Preparing the rayon fibers samples for mechanical properties determinations.

Section 3. Fixation of the staple fibers-bundle's rayon in the Universal Testing Machine.

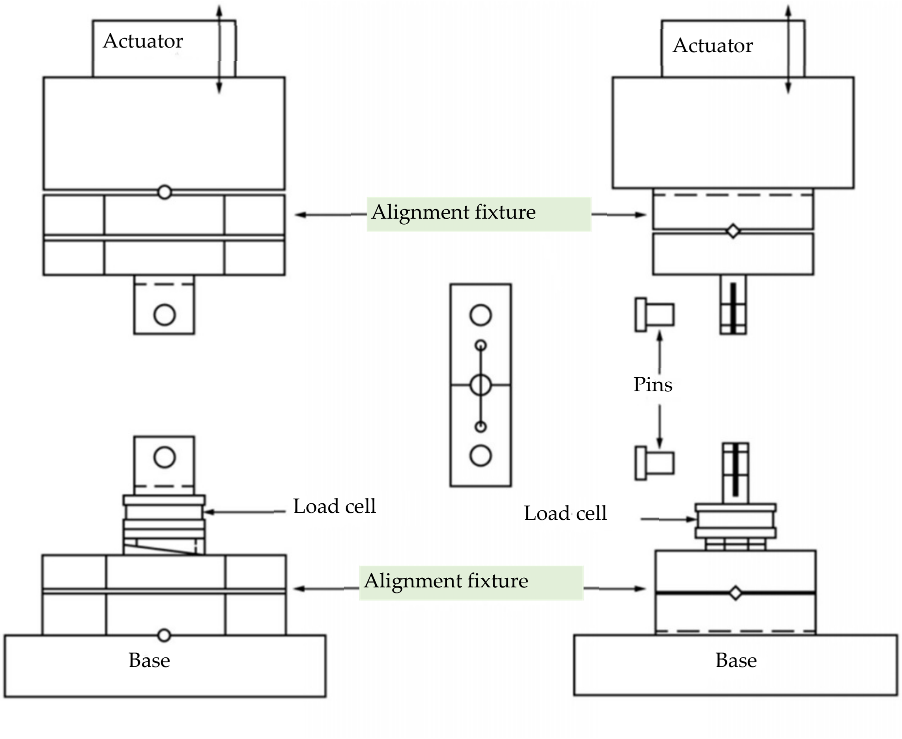

Figure S3. Schematic representative of fixing the staple fibers-bundle's rayon for measuring the tensile properties.
